# Suppression of the alternative lengthening of telomere pathway by the chromatin remodelling factor ATRX

**DOI:** 10.1038/ncomms8538

**Published:** 2015-07-06

**Authors:** David Clynes, Clare Jelinska, Barbara Xella, Helena Ayyub, Caroline Scott, Matthew Mitson, Stephen Taylor, Douglas R. Higgs, Richard J. Gibbons

**Affiliations:** 1 MRC Molecular Haematology Unit, Weatherall Institute of Molecular Medicine, University of Oxford, John Radcliffe Hospital, Oxford OX3 9DS, UK; 2 Computational Biology Research Group, Weatherall Institute of Molecular Medicine, University of Oxford, John Radcliffe Hospital, Oxford OX3 9DS, UK

## Abstract

Fifteen per cent of cancers maintain telomere length independently of telomerase by the homologous recombination (HR)-associated alternative lengthening of telomeres (ALT) pathway. A unifying feature of these tumours are mutations in ATRX. Here we show that expression of ectopic ATRX triggers a suppression of the pathway and telomere shortening. Importantly ATRX-mediated ALT suppression is dependent on the histone chaperone DAXX. Re-expression of ATRX is associated with a reduction in replication fork stalling, a known trigger for HR and loss of MRN from telomeres. A G-quadruplex stabilizer partially reverses the effect of ATRX, inferring ATRX may normally facilitate replication through these sequences that, if they persist, promote ALT. We propose that defective telomere chromatinization through loss of ATRX promotes the persistence of aberrant DNA secondary structures, which in turn present a barrier to DNA replication, leading to replication fork stalling, collapse, HR and subsequent recombination-mediated telomere synthesis in ALT cancers.

## Abstract (web summary)

ATRX, a chromatin remodelling factor, is mutated in cancers that maintain telomere length by alternative lengthening of telomeres (ALT). Here, the authors show that ectopic expression of ATRX triggers telomere shortening, ALT suppression and reduced replication fork stalling.

## Graphical abstract

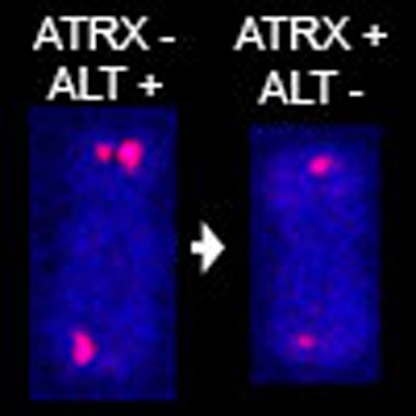

To divide indefinitely, tumour cells must maintain the lengths of their chromosome ends (telomeres), which consist of arrays of G-rich repeats (TTAGGG)_*n*_. In the absence of specialized telomere maintenance mechanisms, linear chromosomes progressively shorten with every round of DNA replication, eventually leading to cellular senescence or apoptosis (reviewed in ref. 1). In most tumours, the maintenance of telomeres is achieved via upregulation of telomerase that adds TTAGGG repeats to telomeres allowing indefinite cell division and tumour progression. However, 15% of cancers achieve immortality via an alternative, telomerase-independent mechanism (the so-called ALT pathway), which depends on homologous recombination (HR)^2^,^3^. The initiation and maintenance of this alternative telomere maintenance pathway in malignancy is poorly understood.

Somatic cell hybridization analyses have previously shown that fusion of ALT cells with normal fibroblasts leads to the inhibition of the ALT pathway indicating that the loss of one or more critical factor(s) triggers ALT^4^. Recently, high throughput genome sequencing of a variety of cancers has identified inactivating mutations in the ATRX/DAXX/H3.3 complex in cells and tumours exhibiting the ALT phenotype, including pancreatic neuroendocrine tumours^5^, glioblastoma multiforme, oligodendrogliomas, medulloblastomas^6^ and neuroblastomas^7^. Although it has been proposed that ATRX is a tumour suppressor, the role of ATRX in ALT has remained unclear. ATRX is a chromatin remodelling factor of the Snf2 family^8^ which, together with the histone chaperone DAXX, facilitates the incorporation of the histone variant H3.3 into telomeric^9^,^10^ and pericentromeric chromatin^11^. The recent finding that co-depletion of the histone chaperones ASF1a and ASF1b leads to manifestation of the ALT phenotype strongly infers that perturbations in the chromatin environment at telomeres may be important in the engagement of the ALT phenotype^12^. In contrast to ATRX/DAXX, however, ASF1 is expressed in ALT cell lines and ASF1 mutations have yet to be identified in any ALT cancers. Understanding the mechanism by which the ATRX/DAXX complex suppresses the ALT pathway, therefore, remains of paramount importance.

Important recent studies have shown that ATRX binds G-rich repeats (including telomere repeats) that may form non-B DNA structures *in vivo*, including G-quadruplex (G4) structures^13^. Significantly, the finding that *Atrx*-null neuroprogenitor cells are hypersensitive to the G4-stabilizing ligand telomestatin suggests that ATRX may help overcome the effect of such structures^14^, although it is not known what role ATRX might play in this. It is now evident that the presence of G4 structures in the genome likely presents a barrier to multiple nuclear processes, including DNA replication. Indeed G4-stabilizing ligands are known to induce replicative stress, leading to DNA damage at sites in the vicinity of G4-forming sequences^15^,^16^. Loss of functional ATRX has recently been linked to similar replicative stress responses^10^,^14^,^17^,^18^,^19^, lending further credence to a role for ATRX in facilitating the replication of these sequences. The recent finding that in the absence of ATRX replication protein A (RPA) persists at telomeres following DNA replication^20^, and that the ALT pathway is dependent on ATR-dependent sensing of replicative stress^12^ suggest that these functions of ATRX are likely relevant in the suppression of ALT.

Furthermore, it was recently shown that ATRX interacts with components of the MRE11-RAD50-NBS1 (MRN) complex, which is known to play key roles in facilitating genomic stability and replication, including the restart of stalled replication forks and the repair of double strand breaks (via both HR and non-homologous end joining). Critically, depletion^21^ or sequestration^22^ of MRN inhibits the ALT pathway raising the possibility that ATRX may also influence ALT by modulating MRN activity in some capacity.

Here we investigate the relationship between ATRX expression and the ALT phenotype in U-2 OS cells, which represent one of the best-characterized models for dissecting this pathway in cancer cells. We show that ectopic expression of ATRX leads to a rapid decline in the cardinal hallmarks of ALT^23^; telomere sister chromatid exchange (TSCE), ALT-associated promyelocytic leukaemia (PML) nuclear bodies (APBs) and extrachromosomal C-circles, with concomitant telomere shortening. We also provide evidence that ATRX plays a central role in suppressing ALT via apparently two converging pathways. First, we show that ATRX-mediated suppression of ALT is dependent on the histone H3.3 chaperone DAXX and an increase in telomeric levels of histone H3.3. Moreover, this suppression is associated with a decrease in replication fork stalling, likely owing to increased telomere chromatinization limiting the formation of G4 structures, which form a barrier to the replication fork and a substrate for HR. In line with this, we find that stabilization of G4 structures potentiates ALT in the presence of ATRX. Second, ATRX interacts with and sequesters the MRN complex that, in the absence of ATRX, is redistributed to APB sites where it may facilitate HR. In this report, we link the induction of ALT with the presence of a DNA secondary structure and this may also explain why ATRX is being increasingly identified as a tumour suppressor in a wide variety of ALT-positive tumours.

## Results

### Expressing ATRX in U-2 OS cells reverses the ALT phenotype

**Figure 1:**
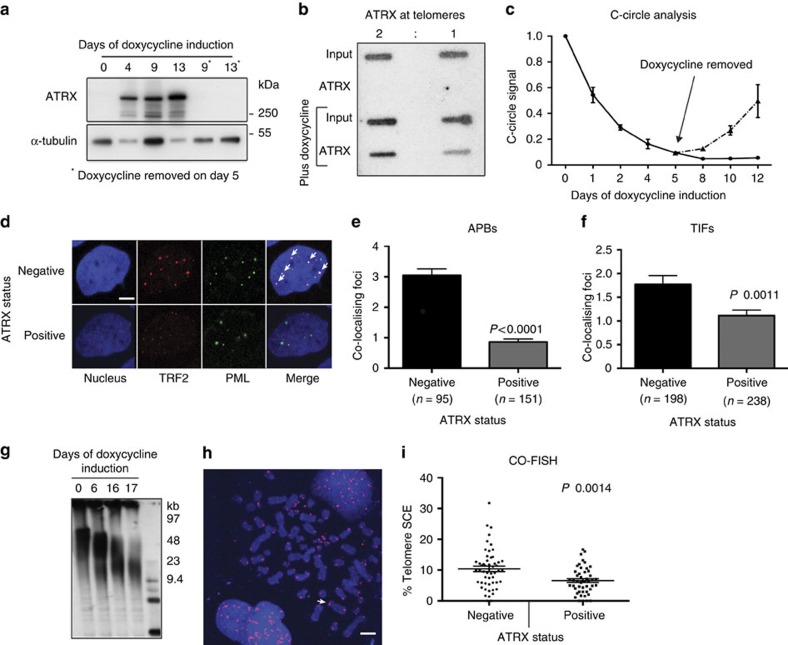
Exogenously expressed ATRX represses the ALT pathway. (**a**) Immunoblot showing expression of ATRX in U-2 OS^*ATRX*^ cell line on addition of 0.4 μg ml^−1^ doxycycline for the specified number of days. Alpha tubulin is shown as a loading control. (**b**) Chromatin Immunoprecipitation showing that on addition of 0.4 μg ml^−1^ doxycycline for 4 days the exogenously expressed ATRX is targeted to telomeres. Immunoprecipitated DNA was slot blotted and probed with a ^32^P-labelled TTAGGG probe. (**c**) Induction of ATRX by doxycycline leads to a fall in C-circles which is reversed on withdrawal of doxycycline at day 5 (shown as a dashed line). (**d**) Representative immunofluorescence images showing the presence of ALT-associated PML bodies (APBs) in untreated U-2 OS^*ATRX*^ cells and the reduction in APBs on induction of ATRX expression by addition of 0.4 μg ml^−1^ doxycycline for 4 days. A 10-μm scale marker is shown. APBs (**e**) and TIFs (**f**) are scored as a direct co-localization between either TRF2 and PML for APBs, or between TRF2 and 53BP1 for TIFs, with the chart showing the average number of APBs or TIFs in U-2 OS^*ATRX*^ cells with and without ATRX expression. Co-localizing foci were scored using the JACoP plugin for ImageJ. Number of cells scored (*n*) is shown in parentheses. Statistical significance was determined using a Mann–Whitney test. (**g**) Terminal restriction fragment length analysis using a TTAGGG probe showing progressive telomere shortening on expression of ATRX. (**h**) Representative image of chromosome orientation fluoresence *in situ* hybridization (CO-FISH). G-rich telomeric DNA is stained in red. A crossover event is depicted with a white arrow, a 10-μm scale marker is shown. (**i**) Quantitation of CO-FISH in U-2 OS^*ATRX*^cells before and after ATRX induction for 4 days. Telomere sister chromatid exchange (TSCE) was scored as a percentage of exchanges per metaphase spread. Error bars denote s.e.m. Over 2,000 telomere ends were scored for cross-overs per treatment group. Statistical significance was determined using a Mann–Whitney test.

Mutations in the chromatin remodeller ATRX have been identified in many ALT-associated cancers and in a comprehensive study it was found that 90% of ALT-immortalized cell lines lack ATRX^24^. To date, however, it is unclear as to whether mutations in ATRX act as a driver or passenger in the formation of such neoplasms. To address this question we re-introduced ATRX into telomerase negative, U-2 OS, ALT cells, which are null for ATRX because of a homozygous deletion in the *ATRX* gene^6^. We stably transfected U-2 OS cells with an ATRX cDNA under the control of a Tet promoter, allowing for doxycycline inducible expression of the ATRX protein, which is then rapidly depleted on removal of doxycycline (Fig. 1a, Supplementary Fig. 1a,b). The cell line thus generated will be referred to throughout the text as U-2 OS^*ATRX*^. We sought to gauge the levels of ectopically expressed ATRX relative to endogenous ATRX in the ATRX-positive cell lines 293T and HT1080. Levels of ectopically expressed ATRX were marginally higher, following 4 days of doxycycline treatment, relative to endogenous ATRX in the HT1080 cell line. Importantly, however, the 293T cell line exhibited markedly higher levels of endogenous ATRX (Supplementary Fig. 1c). Taken together, we conclude that the expression level of ectopic ATRX falls within the physiological range for endogenous ATRX observed across cell lines. Consistent with a putative role for ATRX in telomere homeostasis, chromatin immunoprecipitation followed by slot blotting and probing with a telomeric probe confirmed that ectopically expressed ATRX bound to telomeric DNA in U-2 OS^*ATRX*^ cells on doxycycline treatment (Fig. 1b). Moreover, ATRX was clearly detectable by immunofluorescence in the nucleus of U-2 OS^*ATRX*^ cells on doxycycline treatment, and found to extensively co-localize with the shelterin component TRF2 (Supplementary Fig. 1d). ATRX re-expression in U-2 OS^*ATRX*^ cells resulted in a small growth defect (Supplementary Fig. 1e), although no gross defects in cell cycle were evident (Supplementary Fig. 1f). We do note, however, that ATRX re-expression is associated with a small, but reproducible, increase in the percentage of cells in S-phase (mean of 45% and 51% in ATRX-negative and -positive cells, respectively) and a concomitant decrease of cells in G2/M (Supplementary Fig. 1g). Furthermore, no increases in apoptosis were observed on ectopic ATRX expression for 4 days (Supplementary Fig. 1h).

We next determined whether ectopic expression of ATRX suppressed the ALT pathway by monitoring cardinal features of the ALT phenotype. C-circles are partially double stranded extrachromosomal telomeric sequences, which are thought to be intermediates of ALT, generated via telomeric HR. C-circles are considered to be a reliable indicator of ALT behaviour^25^. Inducing expression of ATRX led to a progressive reduction in C-circles, and subsequent silencing of ATRX expression led to their increase (Fig. 1c). No reduction in C-circles was observed on doxycycline treatment of an untransfected U-2 OS cell line, confirming that the observed loss of C-circles is attributable to the expression of ectopic ATRX in U-2 OS^*ATRX*^ cells (Supplementary Fig. 2a). Furthermore, this re-expression of ATRX was associated with a significant reduction in the number of APBs (Fig. 1d,e), which are characteristic intra-nuclear bodies seen in the ALT phenotype. APBs contain PML, telomeric DNA and proteins associated with telomeres, DNA replication, recombination and repair. Comparable results were obtained with an independently derived clone (Supplementary Fig. 2b,c).

The telomeres of ALT cells are characterized by the persistent presence of DNA damage foci, termed telomere dysfunction induced foci (TIFs). In accordance with a suppression of the ALT pathway, re-expression of ATRX in U-2 OS^*ATRX*^ cells was associated with a significant decrease in TIFs, as detected by a loss of co-localization between TRF2 and 53BP1 foci (Fig. 1f)

In the absence of telomerase, the ALT pathway maintains telomere length and enables cell growth. Consistent with a role for ATRX in suppressing this pathway, on ATRX expression in the U-2 OS^*ATRX*^ cell line we observed a progressive decrease in the length of telomeres as assessed by telomere restriction fragment (TRF) analysis (Fig. 1g, Supplementary Fig. 2d). Moreover, removal of doxycycline led to a progressive recovery in telomere length, reflecting a reinitiation of the ALT pathway on ATRX removal (Supplementary Fig. 2d). Finally, to determine whether ectopic ATRX expression affected the recombinogenic state of the telomeres, TSCE was assessed using chromosome orientation fluoresence *in situ* hybridization (CO-FISH). Consistent with a repression of ALT, ectopic expression of ATRX was associated with a significant reduction in TSCE (Fig. 1h,i)

We next asked whether this ATRX-mediated suppression of the ALT pathway is specific to U-2 OS cells or a general feature of all ALT cancers. Therefore, we transfected another ALT-immortalized cell line, Sa-OS2, previously reported to lack detectable ATRX protein^24^ with an ATRX cDNA. We were unable to generate any stable clones expressing ectopic ATRX. Nonetheless, APBs were markedly reduced following transient transfection of the ATRX cDNA for 48 h (Supplementary Fig. 2e), strongly suggesting that ATRX functions as a tumour suppressor in other ALT cancers. Taken together, it is apparent that despite being insufficient to trigger ALT^17^, loss of functional ATRX is critically required for telomere maintenance via the ALT pathway.

### Suppression of ALT depends on the histone chaperone DAXX

**Figure 2:**
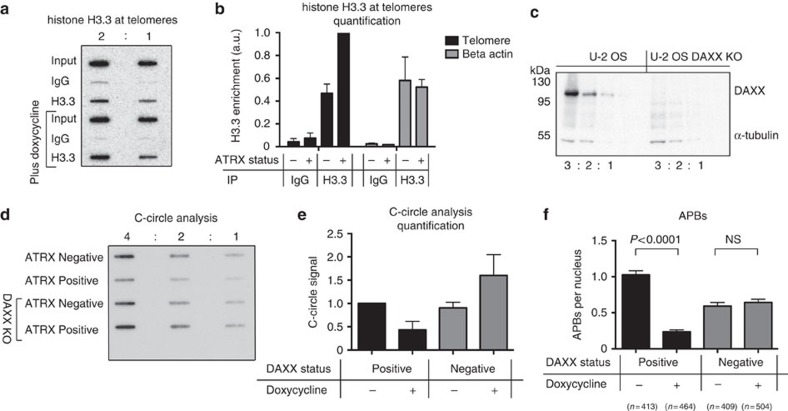
Re-introduction of ATRX increases levels of telomeric histone H3.3. (**a**) Representative blot showing H3.3 ChIP in the absence or presence of ATRX (following 4 days treatment with 0.4 μg ml^−1^ doxycycline). Immunoprecipitated DNA was slot blotted and probed with a TTAGGG probe. (**b**) Graph showing average quantitation of H3.3 ChIP assays from three biological replicates. Levels of H3.3 at beta-actin were assessed by qPCR and did not change on re-expression of ATRX. Error bars indicate ±s.e.m. Enrichment is shown relative to ATRX-positive telomeric histone H3.3 levels. (**c**) Immunoblot showing serial dilutions of a U-2 OS^*ATRX*^ clone in which DAXX was successfully knocked out. Alpha tubulin is shown as a loading control. (**d**) Slot blot of a representative C-circle assay in U-2 OS^*ATRX*^ and DAXX null clone in the absence or presence of ATRX (induced for 4 days). Serial dilutions are shown. Quantification of three biological replicates is shown in (**e**). (**f**) Quantification of three biological replicates of APB immunofluorescence in U-2 OS^*ATRX*^and DAXX null cells before and after ATRX induction for 4 days. Co-localizing foci were analysed as indicated in Fig. 1d. Statistical significance was determined using a Mann–Whitney test.

We next sought to understand how ATRX suppresses the ALT pathway. It has previously been shown that deposition of histone H3.3 at telomeres in mouse ES cells normally depends on a protein complex containing ATRX, H3.3 and the histone chaperone DAXX^9^,^26^. ATRX/DAXX mediated nucleosome assembly at telomeres may stabilize DNA in a duplex configuration^27^, disfavouring non-B-form, HR inducing DNA structures thereby suppressing the ALT pathway or, like HIRA, it may be important in re-establishing chromatin organization after replicative stress by chromatin priming^28^ or nucleosomal gap filling^29^. The identification of DAXX and H3.3 mutations in certain ALT tumours suggests that this pathway is indeed important in the suppression of ALT. We initially asked whether re-expression of ATRX could alter telomeric H3.3 levels in the U-2 OS^*ATRX*^ cell line. Chromatin immunoprecipitation (ChIP) analysis was performed using an antibody that specifically identifies histone H3.3 and showed strikingly that the levels of telomeric H3.3 were increased approximately twofold on ectopic expression of ATRX for 4 days with no change at beta-actin, GAPDH or the sub telomeric region of chromosome 16 (Fig. 2a,b, Supplementary Fig. 3a). We next determined whether this ATRX-mediated deposition of telomeric H3.3 was functionally important in the ATRX-mediated suppression of the ALT pathway. ATRX-targeted deposition of histone H3.3 has previously been shown to be dependent on its association with DAXX^26^, and consistent with this DAXX could be readily immunoprecipitated from U-2 OS^*ATRX*^ nuclear extract on doxycycline treatment, using an ATRX-specific antibody (Supplementary Fig. 3b). We reasoned that if the ATRX-induced suppression of ALT was dependent on the DAXX/H3.3 pathway, then ablation of DAXX from the U-2 OS^*ATRX*^ cell line should negate the ability of ATRX to suppress ALT. To this end we used a genome-editing approach using zinc finger endonucleases to knockout genomic *DAXX*. One *DAXX* knockout clone was derived and successful depletion of DAXX, along with inducible ATRX expression, was confirmed by western blotting analysis (Fig. 2c, Supplementary Fig. 3c,d). Ion torrent sequencing revealed the presence of two heterozygous genomic insertions (GAGC or GAGCC) immediately adjacent to the zinc finger endonucleases cut site (hg19 Chr6 33288596) (Supplementary Fig. 3e). As expected, the increased accumulation of telomeric histone H3.3 was no longer observed upon ATRX expression in the absence of DAXX (Supplementary Fig. 3f). The capacity for ATRX to suppress the ALT pathway was once more assessed using both the C-circle assay and via the detection of APBs. Strikingly the ectopic expression of ATRX was no longer associated with a decrease in the level of C-circles or APBs, as compared with the untreated U-2 OS^*ATRX*^ cell line (Fig. 2d–f). Taken together, we conclude that the ATRX-mediated suppression of ALT is dependent on the presence of DAXX.

### Stabilizing G4 prevents suppression of ALT by ATRX

**Figure 3:**
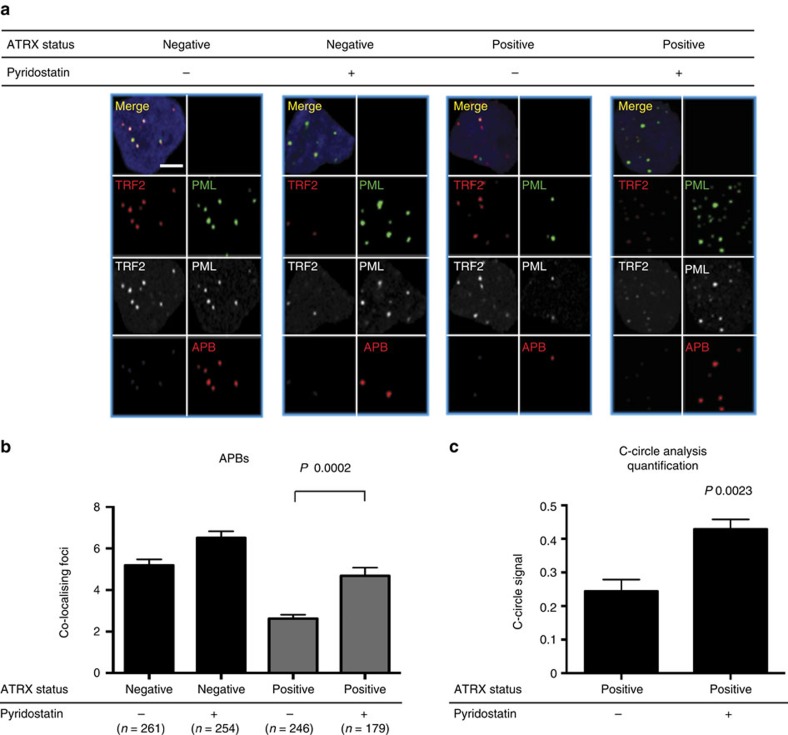
Treatment with a G4-stabilizing ligand impedes ability of ATRX to suppress ALT. Cells were treated for 4 days with 0.4 μg ml^−1^ doxycycline and PDS treatment after day 2 for 2 days (**a**) Representative immunofluoresence images showing output of co-localization analysis between PML and TRF2 as displayed using HTML5 PIVOT software. Row 3 shows foci (displayed in white) called by ImageJ and row 4 shows the number and position of co-localizing foci, representing APBs. A 10-μm scale marker is shown. (**b**) Quantitation of APB numbers per nuclei, number of cells scored is shown in parentheses. Statistical significance was determined using a Mann–Whitney test. (**c**) Graph displaying average quantitation of three C-circle assay biological replicates, showing a higher level of C-circles on treatment with PDS in cells treated with 0.4 μg ml^−1^ doxycycline for 4 days. Statistical significance was determined using a Mann–Whitney test. Original representative blot is shown in Supplementary Fig. 4c.

It has been proposed that defective telomeric chromatinization on loss of ATRX function may be permissive for the formation of non-canonical DNA conformations, including G4 (ref. 27). G4s are thought to provide a barrier to nuclear processes, including transcription and DNA replication. As a result, such structures may promote aberrant homology directed repair (HDR; as occurs in the ALT phenotype) and thereby potentially enhance telomere maintenance via this pathway. Pyridostatin (PDS) is a highly selective G4-interacting ligand, which is known to induce telomere dysfunction^16^. We predicted that if ATRX normally maintains DNA in a B-form conformation, stabilization of G4 by PDS could interfere with the capacity of ATRX to suppress the ALT pathway. Consistent with our previous observations (Fig. 1e) in untreated U-2 OS^*ATRX*^cells expression of ectopic ATRX led to a significant decrease in APB levels. By contrast we found that in the presence of PDS, when ATRX is expressed, APBs are diminished, but not reduced to levels seen in the absence of PDS (Fig. 3a,b, Supplementary Fig. 4a). Treatment of the ALT-negative HeLa cell line with PDS failed to induce the *de novo* formation of APBs (Supplementary Fig. 4b), demonstrating that treatment with PDS does not induce the formation of APBs *per* se but rather prevents the loss of APBs on ATRX expression. C-circle levels also reflected this pattern with significantly higher levels of C-circles observed on PDS treatment in the presence of ATRX (Fig. 3c, Supplementary Fig. 4c). Thus it appears that the presence of G4 structures increases ALT activity and stabilization of telomeric G4 by PDS diminishes the ability of ATRX to overcome this effect.

### ATRX expression leads to reduced replication fork stalling

**Figure 4:**
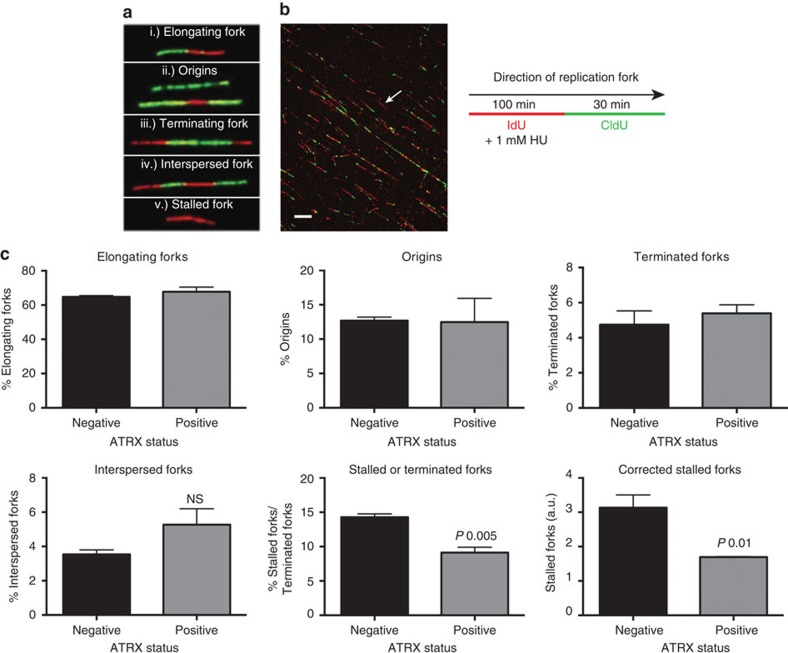
Exogenously expressed ATRX leads to a reduction in replication fork stalling. (**a**) Representative images of five classes of replication intermediates identified in this study. (**b**) Representative image of actual fibres from U-2 OS^*ATRX*^ cells with a white arrow indicating a stalled fork, a 10 μm scale marker is shown. (**c**) Relative frequency of replication intermediates in ATRX-negative and ATRX-positive (4 days treatment with 0.4 μg/ml doxycycline) U-2 OS^*ATRX*^ cells. Over 1,400 fibres totalled from three independent replicates were scored and error bars indicate±s.e.m. Statistical significance was determined using a Mann–Whitney test. Stalled forks were calculated as a ratio of the % stalled or terminated forks and terminated forks.

APBs and C-circles have recently been shown to arise from ATR-dependent sensing of replicative stress, suggesting that HDR of stalled replication forks at telomeres is fundamental in the ALT pathway^12^. We and others have previously shown that the loss of ATRX is associated with increased replication fork stalling and replicative stress in a variety of cell types^14^,^17^,^18^,^19^. It therefore seems likely that increased fork stalling, perhaps resulting from telomeric secondary structures, such as G4, contributes to the initiation and/or maintenance of the ALT phenotype in the absence of ATRX. To test this hypothesis we used DNA fibre analysis to study replication intermediates at single-molecule resolution in the U-2 OS^*ATRX*^ cell line both in the absence and presence of ectopically expressed ATRX. This technique involves labelling replicating cells sequentially with nucleotide analogues (iododeoxyuridine (IdU) and chlorodeoxyuridine (CldU)), cells are then lysed and the DNA fibres spread on glass slides, probed with specific antibodies and visualized by fluorescence microscopy^30^,^31^,^32^. This allows for the identification of five major replication intermediates (Fig. 4a,b). Stalled or terminated replication forks were identified as single ldU labelled (red) fibres where the fork had either stalled or terminated during the first (red) pulse and was therefore refractory to labelling during the second (green) pulse (Fig. 4av). Termination events that occur during the second CldU pulse can also be identified as two elongating forks colliding during the second CldU (green) pulse (Fig. 4aiii). The degree of fork stalling could then be determined as a ratio between the frequency of intermediates v and iii. We found that the frequency of stalled replication forks is consistently reduced on ectopic ATRX expression (Fig. 4c) with no significant changes detectable in the other intermediates. It is apparent then that the loss of ATRX in an ALT tumour cell line leads to an increase in replication fork stalling. Although we have not formally shown that these stalled forks are at telomeres, it is likely that the telomeres represent a site of increased replicative stress on the loss of ATRX, as evidenced by an increase in DNA damage-associated TIFs^10^,^14^,^17^, which in turn triggers HDR and the ALT phenotype.

### ATRX sequesters MRN complex away from telomeres and APBs

**Figure 5:**
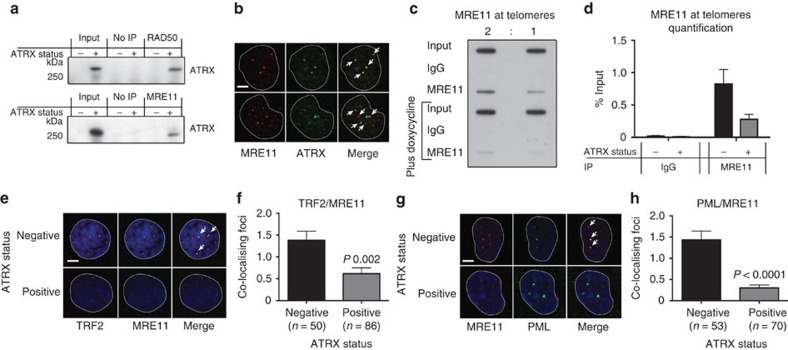
Exogenously expressed ATRX in U-2 OS^*ATRX*^ cells interacts with the MRN complex and alters its localization. (**a**) Nuclear extracts from U-2 OS^*ATRX*^ cell line with and without exogenously expressed ATRX were immunoprecipitated with both RAD50 and MRE11 antibodies. For no IP beads blocked beads were added without prior antibody coupling. The presence of ATRX in immunoisolated interaction partners was assessed by western blotting. (**b**) Immunofluorescence with white arrows showing co-localization between exogenously expressed ATRX and MRE11 in the U-2 OS^*ATRX*^ cell line in two representative nuclei. A 10-μm scale marker is shown. (**c**) Representative blot showing MRE11 ChIP in both the absence and presence of ATRX (following 4 days treatment with 0.4 μg ml^−1^ doxycycline). Immunoprecipitated DNA was slot blotted and probed with a ^32^P-labelled TTAGGG probe. (**d**) Graph showing average quantitation of MRE11 at telomeres by ChIP assay from three biological replicate. Error bars indicate ±s.e.m. (**e**,**f**) Immunofluorescence showing loss in co-localization (white arrows) between MRE11 and telomeric marker TRF2 or (**g**,**h**) MRE11 and PML on expression of ATRX (13 days treatment with 0.4 μg ml^−1^ doxycycline). Number of cells scored are shown in parentheses. Statistical significance was determined using a Mann–Whitney test. A 10-μm scale marker is shown.

HDR and restart of stalled/collapsed replication forks is dependent on the MRN complex which facilitates 5′ to 3′ resection of DNA ends to create 3′ overhangs for strand invasion and in turn is known to be required for ALT activity. We have previously demonstrated that ATRX interacts with the MRN complex in HeLa nuclear extracts^17^ and this led us to consider whether an interaction between ATRX and MRN may also play a role in ATRX-mediated ALT suppression. To address this we prepared nuclear extracts from U-2 OS^*ATRX*^ cells both in the presence and absence of ectopically expressed ATRX. Consistent with such an interaction we found that ATRX could be immunoprecipitated using previously characterized antibodies^17^ against RAD50 and MRE11 only on doxycycline treatment (Fig. 5a, Supplementary Fig. 5a,b). Moreover, we detected a clear co-localization between ATRX and MRE11 by immunofluorescence (Fig. 5b). We next tested the effect of ATRX expression on the distribution of the MRN complex in U-2 OS^*ATRX*^ ALT cells. Consistent with a role for ATRX in inhibiting MRN-mediated telomeric recombination, ChIP analysis showed that the re-expression of ATRX was associated with a marked loss of telomeric associated MRE11 (Fig. 5c,d). Moreover, a significant loss of co-localization between MRE11 and the shelterin components TRF1 and TRF2 was observed on ATRX expression (Fig. 5e,f, Supplementary Fig. 5c–e). It is widely considered that APBs constitute an assembly site for the telomeric recombination machinery in ALT tumours and consistent with this, sequestration of MRN components away from PML is known to suppress the ALT pathway^22^. We therefore reasoned that ATRX may act to sequester the MRN complex from PML and the telomeric machinery, thereby limiting telomeric HDR. Accordingly, we observed a marked loss in the association of MRE11 with PML on ATRX expression (Fig. 5g,h, Supplementary Fig. 5f). As expected, we note that the co-localization between MRE11 and ATRX was generally distinct from PML, with 88% of ATRX/MRE11 co-localizing in foci that were separate from PML (Supplementary Fig. 5g,h). Our data therefore suggest that ATRX actively sequesters the MRN complex, which is a critical component of the ALT pathway, at sites distinct from PML and telomeres and this is associated with the disassembly of APBs and suppression of telomeric HDR.

## Discussion

Recent genetic data derived from sequencing of a wide variety of tumours have correlated mutations in the ATRX/DAXX/H3.3 complex with expression of the ALT pathway but to date it was unclear as to whether these mutations were drivers or merely passengers during tumorigenesis, with no mechanistic connection yet made. Here we show that ATRX acts as a *bona fide* suppressor of the ALT pathway. Re-expression of ATRX in the telomerase negative, ALT-positive cell line U-2 OS led to a marked loss of the cardinal hallmarks of the ALT pathway (APBs and extrachromosomal C-circles and TSCE with concomitant telomere shortening). This decline is rapid with a substantial loss of C-circles detectable within 24 h of ATRX expression. Moreover, this effect is fully reversible with a similarly rapid gain in C-circles and telomere length on switching off ATRX expression. These data demonstrate that gain of ATRX alone is sufficient to fully repress the ALT pathway in this cellular context. Transient transfection of ATRX cDNA into a further ALT-immortalized cell line, Sa-OS2 led to a similar suppression in APBs. Given that a lack of detectable ATRX has been reported in 90% of ALT-positive immortalized cell lines^24^ this suggests that the loss of this tumour suppressor is responsible for ALT in many of these cancers.

We additionally provide mechanistic insights to suggest how ATRX suppresses the ALT pathway. The recent findings that ALT telomeres are characterized by a reduced nucleosome density^33^, and that co-depletion of the histone chaperones ASF1a and ASF1b lead to the engagement of various ALT-associated features^12^, implies that histone deposition may play a central role in normally restricting the recombinogenic potential of telomeres. Consistent with this, we report that the ectopic expression of ATRX is associated with a marked increase in telomeric histone H3.3 levels and, in agreement with previous reports^11^,^26^ this deposition was dependent on the histone chaperone DAXX. Importantly, the ability of ATRX to suppress the ALT pathway was also dependent on the presence of DAXX, strongly inferring that the ATRX/DAXX H3.3 deposition pathway is fundamental in the repression of ALT. Indeed mutations in DAXX have been identified in ALT-positive pancreatic neuroendocrine tumours, paediatric gliobastoma multiforme and selected ALT-immortalized cell lines^6^,^24^,^34^, underlying their importance in the suppression of the ALT pathway. It is of note, however, that the frequency of ATRX mutations identified in ALT tumours and cell lines appears markedly more prevalent than those identified for DAXX, with no DAXX mutations yet identified in oligodendriglomas, medulloblastomas and several ALT-immortalized cell lines^6^,^24^. A parsimonious explanation for this is that ATRX is simply more prone to mutation than DAXX, but alternatively it may suggest that ATRX has additional DAXX/H3.3 pathway independent roles in the suppression of ALT.

A potential consequence of defective telomere chromatinisation on loss of ATRX function is an increased propensity to form non-canonical DNA secondary structures, such as G4 (ref. 27). Here we show that treatment with the G4-stabilizing ligand PDS leads to the ALT pathway becoming partially resistant to suppression by ATRX. This suggests that the presence of such structures at telomeres potentiates ALT and that ATRX normally plays a role in overcoming their deleterious effect. The recent finding that *Atrx*-null neuroprogenitor cells exhibit hypersensitivity to the G4-stabilizing ligand telomestatin further supports this model^14^. We have, however, previously shown that ATRX itself does not appear to possess G4-unwinding activity^17^, suggesting that ATRX plays an indirect role in overcoming these impediments. Telomeres have emerged as fragile sites, regions of the genome where replication fork collapse and restart by HDR occur at elevated frequencies^35^. It is likely that the presence of G4 structures in telomeric DNA facilitates the ALT phenotype by presenting a barrier to the replication fork, triggering fork stalling, collapse and subsequent restart by HDR. In accordance with this, PDS has previously been shown to induce replication dependent DNA damage, with aphidicolin-facilitated arrest of S-phase leading to a marked loss of γH2AX foci in PDS-treated cells^16^. Furthermore, treatment with another G4-interacting ligand RHPS4 has been shown to prohibit telomere replication and trigger an ATR-dependent signalling response^15^. Similar defects have been reported on loss of ATRX function in several cell types, with increases in replication fork stalling, prolongation in S-phase and elevated telomeric DNA damage foci^14^,^17^,^18^,^19^. It is likely then that these defects are attributable to the presence of G4, creating a barrier to fork progression upon loss of ATRX function. G4 structures have been shown to form co-transcriptionally in the context of ‘G-loops’ whereby the G4 structure presents on one strand, adjacent to a stable RNA/DNA hybrid on the other strand^36^. Significantly, levels of the telomeric long non-coding RNA TERRA are elevated in ALT cancer cells and on depletion of ATRX in certain cell lines^9^,^20^,^33^. This raises the possibility that increased TERRA transcription may also contribute to the formation of recombinogenic DNA secondary structures on loss of ATRX function. In support of this, overexpression of the RNaseH1 endonuclease, which processes RNA:DNA hybrids, abrogates the recombinogenic potential of telomeres in ALT-positive cells^37^.

There is mounting evidence that the HR-mediated repair of stalled replication forks is a key determinant of the ALT pathway. Depletion of factors required for recombination-mediated fork restart (including MRN, MUS81 and FANCD2), suppress telomeric recombination in ALT cells^38^,^39^. Consistent with this we show that ectopic expression of ATRX in the U-2 OS^*ATRX*^ cell line significantly reduces replication fork stalling, thereby likely limiting the substrate for HDR and downregulating ALT. We additionally show that ectopically expressed ATRX interacts with endogenous MRN complex components in the U-2 OS^*ATRX*^ cell line. HDR of collapsed forks is dependent on several proteins, many of which are critical for ALT, including components of the MRN complex. MRN catalyses the 5′ to 3′ resection of DNA ends, creating a 3′ overhang that is necessary for subsequent strand invasion. The expression of ATRX results in a dramatic redistribution of the MRN complex away from telomeric DNA and PML nuclear bodies. The association of MRN with PML bodies is a known requirement of ALT activity and likely constitutes the sites of telomeric recombination^3^,^40^. Significantly, overexpression of another PML nuclear body component, Sp100, also sequesters MRN components away from APBs, leading to a suppression of ALT^22^. It appears then, that in addition to preventing replicative stress, which serves as an upstream trigger for HDR, ATRX expression may have an analogous effect to Sp100 overexpression, limiting ALT by redistributing MRN away from sites of telomeric recombination. In accordance with this, we find that co-localization of MRE11 and ATRX occurs predominantly at sites distinct from PML. We note that ectopically expressed ATRX is also recruited to telomeres and PML bodies in the U-2 OS^*ATRX*^ cell line and at present it remains unclear how the association between ATRX and MRN occurs specifically at sites distinct from APBs. Interestingly, G4 structures are a preferred substrate of MRN^41^ raising the intriguing possibility that MRN directly cleaves G4 structures that persist in an ALT tumour following DNA replication or transcription, triggering double strand break formation and HDR.

**Figure 6:**
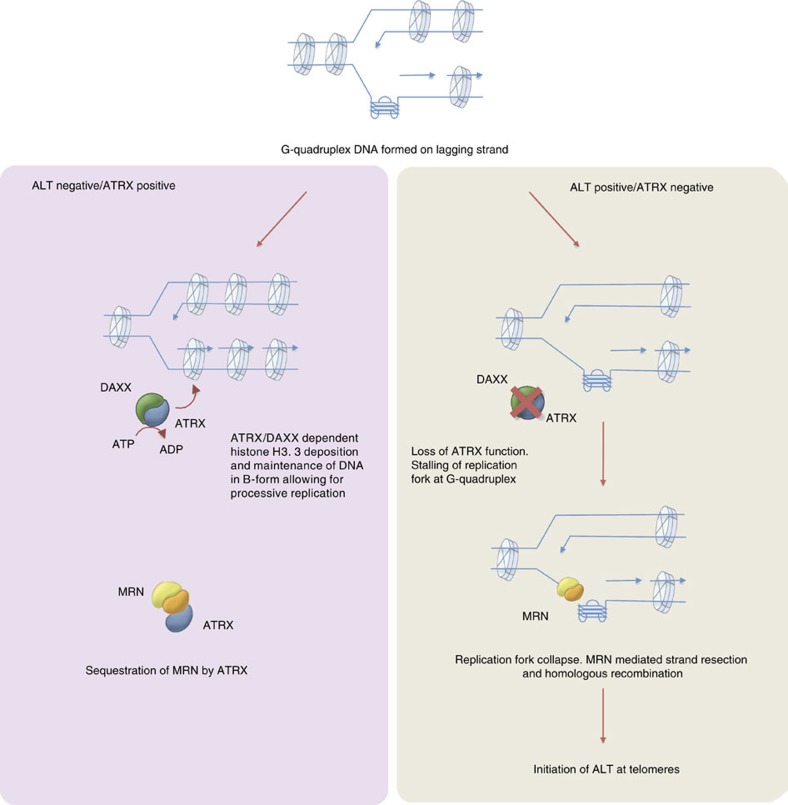
Model for ATRX-mediated suppression of the ALT pathway. ATRX together with DAXX deposits histone H3.3 at telomeres, which in turn may facilitate DNA replication through G-quadruplex sequences. The presence of G-quadruplex structures in an ATRX null tumour cell leads to replication fork stalling and collapse, providing a substrate for MRN-dependent homologous recombination and maintenance of telomere length through ALT. ATRX additionally interacts with the MRN complex, facilitating its distribution away from PML bodies and telomeres, further limiting HR.

Our observations have shown that ATRX plays a central role in ALT cells via apparently two converging pathways. First, without ATRX there is an increase in stalled replication forks, likely owing to the persistence of G4 structures, which promote HR. Second, in the absence of ATRX, redistribution of the MRN complex to APB sites may also facilitate HDR (Fig. 6). These two pathways may function independently, or it is possible that they are part of one pathway linked by the disassembly of APBs. These findings not only provide a mechanistic link between ATRX and the ALT pathway, but may also explain why ATRX is being increasingly identified as a tumour suppressor in a wide variety of ALT-positive mesenchymal tumours. These findings may also provide important clues in the development of future therapies to treat ALT-based cancers. Indeed, one may predict that limiting replicative stress, perhaps through nucleoside supplementation^42^, may be beneficial for the treatment of ALT-based cancers. The finding that a DNA secondary structure potentiates ALT highlights the potential importance of aberrant DNA structure in human pathology.

## Methods

### Expression of ATRX in U-2 OS and Sa-OS2 cells

U-2 OS and Saos-2 cell lines were obtained from ATCC.

ATRX cDNA was cloned into the Tet-on 3G Inducible Expression System (Clontech) and transfected into using Xfect transfection reagent (Clontech), to generate the U-2 OS^*ATRX*^ stable cell line. For Sa-OS2 transient transfections ATRX cDNA was tagged with ZsGreen1 (IRES) using the Lenti-X Tet-On 3G Inducible expression system (Clontech). Co-transfection of 1 μg·pLVX-Tet3G and 4 μg PLVX-TRE3G-ATRX-ZsGreen1 vectors was performed in the presence and absence of 0.4 μg ml^−1^ doxycycline for 48 h using Xfect transfection reagent.

### Knockout of DAXX in U-2 OS^*ATRX*^ cell line

DAXX was knocked out using CompoZr^TM^ Knockout Zinc Finger Nucleases (Sigma CKOZFN6935-1KT).

### Immunofluorescence (IF) and western blotting (WB)

U-2 OS^*ATRX*^ cells grown on coverslips for 4 days with or without 0.4 μg ml^−1^ doxycycline were prepared for IF by standard procedures. Cells were prepermeabilized with ice cold 0.5% Triton X-100 for 5 min before fixation with 4% paraformaldehyde. The following antibodies were used for immunostaining: anti-alpha tubulin (1:50,000, Abcam ab7291); anti-ATRX (1:200 for WB; 1:500 for IF, Santa Cruz sc-15408); anti-DAXX (1:500, Sigma D7810); anti-MRE11 (1:200, Abcam ab214); anti-MRE11 (1:100, Calbiochem PC388); anti-RAD50 (1:200, Abcam ab89); anti-PML (1:200, Santa Cruz sc5621); anti-TRF2 (1:200, Imgenex IMG-124A) and anti-TRF1 (1:50, Santa Cruz sc-1977). For cell cycle analysis anti-BrdU (Abcam ab6326) was used at 1 μg ml^−1^. Secondary antibodies for IF and for cell cycle analysis (Invitrogen Alexa-fluor conjugated) were used at 1:3,000. Secondary antibodies for western blots (Sigma anti-mouse IgG A4416 or Sigma anti-rabbit IgG A6667) were used at 1:10,000. Cells were treated for 48 h with 2 μM PDS (kind gift from Shankar Balasubramanian) and 0.4 μg ml^−1^ doxycycline for the specified number of days. All images were taken on an Olympus BX51 confocal microscope at 100 × magnification. Uncropped images are shown in the Supplementary Information.

### C-circle assay

C-circle assay was based on a previously described protocol^25^. Genomic DNA extracted from U-2 OS^*ATRX*^ cells was treated with RNase A and digested with 1 U Hinf1 and Rsa1. One-hundred and fifty nanograms of digested DNA was combined with 0.1 mg ml^−1^ BSA, 0.05% Tween, 1 mM each dATP, dGTP and dTTP, 1 × Φ29 buffer and 37.5 U Φ29 DNA polymerase (NEB) and incubated for 8 h at 30 °C and then at 65 °C for 20 min. The reaction products were subsequently diluted in 2 × SSC and slot blotted onto a 2 × SSC soaked Zeta-Probe Blotting membrane (Bio-Rad), which was then hybridized overnight at 37 °C with end-labelled ^32^P-(CCCTAA)_3_ probe in PerfectHyb Plus hybridization buffer (Sigma). Quantification was preformed using ImageJ software and is presented normalized to the C-circle level with no doxycycline treatment (ATRX negative). Cells were treated for 48 h with 20 μM PDS (Sigma) and 0.4 μg ml^−1^ doxycycline for the specified number of days.

### Terminal restriction fragment analysis

Telomere length of uninduced and induced (grown in the presence of 0.4 μg ml^−1^ doxycycline for 6, 16 and 17 days) U-2 OS^*ATRX*^ cells was determined by terminal restriction length analysis^43^. Five micrograms of high-molecular-weight DNA was digested with HinfI and RsaI then separated by pulse-field gel electrophoresis in 1% agarose and 0.5 × TBE at 6 V cm^−1^ for 15 h with switch times of 0.1–6.0 s. After blotting the filter was probed with a ^32^P-labelled TTAGGG probe.

### CO-FISH

CO-FISH was performed using the protocol described by Ourliac–Garnier and Londoño–Vallejo^44^. U-2 OS^*ATRX*^ cells were grown for 4 days with or without 0.4 μg ml^−1^ doxycycline and incubated with BrdU for 19 h before addition of colcemid for an additional 2 h. Cells were collected by trypsinization and incubated 15 min in sodium citrate buffer at 37 °C, fixed with 3:1 ethanol–acetic acid and metaphase spreads were prepared. The slides were then treated with RNase solution, stained with Hoechst 33258 and then ultrviolet-treated, before being digested with Exonuclease III. Metaphases were then fixated in formaldehyde, digested with pepsin solution, fixated again and then dehydrated with 70, 90, and 100% ethanol. They were then incubated with the first probe solution for 1.5 h after denaturation at 80 °C. Slides were washed with a formamide-based solution and then with PBS, dehydrated again and incubated with the second probe solution for an additional 1.5 h. The same washes were performed and the slides were then dehydrated for the last time, stained with ToPro3 and mounted with DAPI for microscopy.

### DNA fibre analysis

For fibre analysis, U-2 OS^*ATRX*^ cells were grown for 4 days with or without 0.4 μg ml^−1^ doxycycline and incubated with IdU in the presence of 1 mM hydroxyurea for 100 min, followed by a 30-min incubation with 250 μM CldU. Spreading and visualization of fibres was performed as previously described^30^.

### Chromatin Immunoprecipitation

For ChIP of ATRX cells were fixed with 2 mM ethylene glycol bis(succinimidyl succinate) (EGS) (Pierce 26103) for 45 min at room temperature in PBS. Formaldehyde was then added to 1% for 20 min and quenched with 125 mM glycine. Chromatin was sonicated to under 500 bp and lysates were immunoprecipitated using 20 μg anti-ATRX antibody (Santa Cruz sc-15408) antibody. DNA was precipitated with 20 μg carrier glycogen. Histone H3.3 chromatin immunoprecipitations were performed using Millipore chromatin Immunoprecipitation Assay Kit (17-295) as per manufacturer’s instructions and 10 μg anti-histone H3.3 antibody (Millipore 09-838)^45^. Telomere binding was assessed by slot blotting and probing with a ^32^P-labelled TTAGGG probe. Binding at other loci was assessed by quantitative-PCR (qPCR); primers are shown in Supplementary Table 1. Quantification was performed using ImageJ software and presented as a percentage of the input DNA.

### Real-time qPCR

Real-Time qPCR analysis of beta-actin was performed using SYBR green master mix (Applied Biosystems 4309155).

### Immunoisolation of ATRX with RAD50 and MRE11 antibodies

U-2 OS^*ATRX*^ nuclear extracts were prepared using Cellytic Nuclear Extraction kit (Sigma) in the absence of detergent and equilibrated into IP buffer (20 mM HEPES pH 7.4, 1% Triton X-100, 150 mM NaCl, 1 mM EDTA, 1 mM EGTA+protease inhibitor cocktail). Immunoprecipitations were performed overnight at 4 °C using 10 μg anti-RAD50 antibody (Abcam ab89) or 10 μg anti-MRE11 antibody (Abcam ab214) with protein G dynabeads. Beads were washed four times in IP buffer and bound proteins eluted into SDS loading buffer (Laemmli) by heating at 90 °C for 5 min. Western blotting was performed using anti-ATRX antibody (Santa Cruz sc-15408).

### Bioinformatics analysis

Image analysis was performed using custom Perl processing scripts that called various ImageJ macros to allow filtering and automatic thresholding methods to segment nuclei and then identify foci per nucleus. The JACoP^46^ plugin was used to examine coincident co-localization. The results were then parsed using Perl scripts and a montage was automatically generated that included the segmented nuclei, auto thresholded images for each channel, original grey scale images for each channel and the JACoP DBC and CC generated image. The images were then made into set of DeepZoom tiling images using the OpenZoom Python library (http://openzoom.org) and an xml data file created and viewed using a HTML5 PivotViewer^47^. The entire process was automated and all scripts are available on request. HTML5 PivotViewer allowed the rapid analysis and review of the many hundreds of images generated from these analyses.

The full data sets for Fig. 1 are available at:

http://zegami.molbiol.ox.ac.uk/collections/TRF2_PML_1/, http://zegami.molbiol.ox.ac.uk/collections/53BP1_Tel2/

http://zegami.molbiol.ox.ac.uk/collections/TRF2_53BP1_1/, http://zegami.molbiol.ox.ac.uk/collections/TRF2_53BP1_2/.

The full data set for Fig. 2 is available at:

http://zegami.molbiol.ox.ac.uk/collections/050115_223_35_APBs_IV/

http://zegami.molbiol.ox.ac.uk/collections/231014_223_Clone35_APBs_II/

http://zegami.molbiol.ox.ac.uk/collections/231014_223_Clone35_APBs_III/

Full data sets for Fig. 3 are available at:

http://zegami.molbiol.ox.ac.uk/collections/TRF2_PML_Dox_4days_1/, http://zegami.molbiol.ox.ac.uk/collections/TRF2_PML_Dox_4days_2/, http://zegami.molbiol.ox.ac.uk/collections/TRF2_PML_Dox_4days_3/.

Full data sets for Fig. 5 are available at:

http://zegami.molbiol.ox.ac.uk/collections/MRE11_TRF2/ http://zegami.molbiol.ox.ac.uk/collections/MRE11_PML_1/.

Full data sets for Supplementary Fig. 5 are available at:

http://zegami.molbiol.ox.ac.uk/collections/TRF2_MRE11_2/, http://zegami.molbiol.ox.ac.uk/collections/MRE11_TRF1_2/. http://zegami.molbiol.ox.ac.uk/collections/MRE11_PML_2/.

http://zegami.molbiol.ox.ac.uk/collections/MRE11_ATRX_PML/

### Cell proliferation assay

Growth rate of U-2 OS^*ATRX*^ cells grown in the presence or absence of 0.4 μg ml^−1^ doxycycline was assessed using the Cell Titer 96®AQeous One Solution Cell Proliferation Assay Kit by Promega. Instructions provided in the kit were followed.

### Apoptosis assay

Apoptosis assay was performed using the Annexin V-FITC Apoptosis Detection Kit (abcam ab14085) as per manufacturer’s instructions.

### Cell cycle analysis

Cells were incubated with 10 μM BrdU for 30 min before collecting. BrdU incorporation and DNA content were assessed by fluorescence-activated cell sorting analysis using propidium iodide staining, anti-BrdU antibody (Abcam ab6326) and goat anti-rat A488 secondary antibody (Invitrogen A11006).

## Additional information

**How to cite this article:** Clynes, D. *et al*. Suppression of the alternative lengthening of telomere pathway by the chromatin remodelling factor ATRX. *Nat. Commun.* 6:7538 doi: 10.1038/ncomms8538 (2015).

## Supplementary Material

Supplementary Information Supplementary Figures 1-5 and Supplementary Table 1

## References

[b1] Gilson E. & Geli V. How telomeres are replicated. Nat. Rev. Mol. Cell Biol. 8, 825–838 (2007). 17885666 10.1038/nrm2259

[b2] Bryan T. M., Englezou A., Dalla-Pozza L., Dunham M. A. & Reddel R. R. Evidence for an alternative mechanism for maintaining telomere length in human tumors and tumor-derived cell lines. Nat. Med. 3, 1271–1274 (1997). 9359704 10.1038/nm1197-1271

[b3] Dunham M. A., Neumann A. A., Fasching C. L. & Reddel R. R. Telomere maintenance by recombination in human cells. Nat. Genet. 26, 447–450 (2000). 11101843 10.1038/82586

[b4] Perrem K. . Repression of an alternative mechanism for lengthening of telomeres in somatic cell hybrids. Oncogene 18, 3383–3390 (1999). 10362359 10.1038/sj.onc.1202752

[b5] Jiao Y. . DAXX/ATRX, MEN1, and mTOR pathway genes are frequently altered in pancreatic neuroendocrine tumors. Science 331, 1199–1203 (2011). 21252315 10.1126/science.1200609 PMC3144496

[b6] Heaphy C. M. . Altered telomeres in tumors with ATRX and DAXX mutations. Science 333, 425 (2011). 21719641 10.1126/science.1207313 PMC3174141

[b7] Molenaar J. J. . Sequencing of neuroblastoma identifies chromothripsis and defects in neuritogenesis genes. Nature 483, 589–593 (2012). 22367537 10.1038/nature10910

[b8] Gibbons R. J., Picketts D. J., Villard L. & Higgs D. R. Mutations in a putative global transcriptional regulator cause X-linked mental retardation with α-thalassemia (ATR-X syndrome). Cell 80, 837–845 (1995). 7697714 10.1016/0092-8674(95)90287-2

[b9] Goldberg A. D. . Distinct factors control histone variant H3.3 localization at specific genomic regions. Cell 140, 678–691 (2010). 20211137 10.1016/j.cell.2010.01.003 PMC2885838

[b10] Wong L. H. . ATRX interacts with H3.3 in maintaining telomere structural integrity in pluripotent embryonic stem cells. Genome Res. 20, 351–360 (2010). 20110566 10.1101/gr.101477.109 PMC2840985

[b11] Drane P., Ouararhni K., Depaux A., Shuaib M. & Hamiche A. The death-associated protein DAXX is a novel histone chaperone involved in the replication-independent deposition of H3.3. Genes Dev. 24, 1253–1265 (2010). 20504901 10.1101/gad.566910 PMC2885661

[b12] O’Sullivan R. J. . Rapid induction of alternative lengthening of telomeres by depletion of the histone chaperone ASF1. Nat. Struct. Mol. Biol. 21, 167–174 (2014). 24413054 10.1038/nsmb.2754 PMC3946341

[b13] Law M. J. . ATR-X Syndrome Protein Targets Tandem Repeats and Influences Allele-Specific Expression in a Size-Dependent Manner. Cell 143, 367–378 (2010). 21029860 10.1016/j.cell.2010.09.023

[b14] Watson L. A. . Atrx deficiency induces telomere dysfunction, endocrine defects, and reduced life span. J. Clin. Invest. 123, 2049–2063 (2013). 23563309 10.1172/JCI65634 PMC3635723

[b15] Rizzo A. . Stabilization of quadruplex DNA perturbs telomere replication leading to the activation of an ATR-dependent ATM signaling pathway. Nucleic Acids Res. 37, 5353–5364 (2009). 19596811 10.1093/nar/gkp582 PMC2760797

[b16] Rodriguez R. . Small-molecule-induced DNA damage identifies alternative DNA structures in human genes. Nat. Chem. Biol. 8, 301–310 (2012). 22306580 10.1038/nchembio.780 PMC3433707

[b17] Clynes D. . ATRX dysfunction induces replication defects in primary mouse cells. PLoS ONE 9, e92915 (2014). 24651726 10.1371/journal.pone.0092915 PMC3961441

[b18] Huh M. S. . Compromised genomic integrity impedes muscle growth after Atrx inactivation. J. Clin. Invest. 122, 4412–4423 (2012). 23114596 10.1172/JCI63765 PMC3533543

[b19] Leung J. W. . Alpha thalassemia/mental retardation syndrome X-linked gene product ATRX is required for proper replication restart and cellular resistance to replication stress. J. Biol. Chem. 288, 6342–6350 (2013). 23329831 10.1074/jbc.M112.411603 PMC3585069

[b20] Flynn R. L. . Alternative lengthening of telomeres renders cancer cells hypersensitive to ATR inhibitors. Science 347, 273–277 (2015). 25593184 10.1126/science.1257216 PMC4358324

[b21] Zhong Z. H. . Disruption of telomere maintenance by depletion of the MRE11/RAD50/NBS1 complex in cells that use alternative lengthening of telomeres. J. Biol. Chem. 282, 29314–29322 (2007). 17693401 10.1074/jbc.M701413200

[b22] Jiang W. Q. . Suppression of alternative lengthening of telomeres by Sp100-mediated sequestration of the MRE11/RAD50/NBS1 complex. Mol. Cell Biol. 25, 2708–2721 (2005). 15767676 10.1128/MCB.25.7.2708-2721.2005 PMC1061646

[b23] Henson J. D. & Reddel R. R. Assaying and investigating alternative lengthening of telomeres activity in human cells and cancers. FEBS Lett. 584, 3800–3811 (2010). 20542034 10.1016/j.febslet.2010.06.009

[b24] Lovejoy C. A. . Loss of ATRX, Genome Instability, and an Altered DNA Damage Response Are Hallmarks of the Alternative Lengthening of Telomeres Pathway. PLoS. Genet. 8, e1002772 (2012). 22829774 10.1371/journal.pgen.1002772 PMC3400581

[b25] Henson J. D. . DNA C-circles are specific and quantifiable markers of alternative-lengthening-of-telomeres activity. Nat. Biotechnol. 27, 1181–1185 (2009). 19935656 10.1038/nbt.1587

[b26] Lewis P. W., Elsaesser S. J., Noh K. M., Stadler S. C. & Allis C. D. Daxx is an H3.3-specific histone chaperone and cooperates with ATRX in replication-independent chromatin assembly at telomeres. Proc. Natl Acad. Sci. USA 107, 14075–14080 (2010). 20651253 10.1073/pnas.1008850107 PMC2922592

[b27] Whitehouse I. & Owen-Hughes T. ATRX: Put me on repeat. Cell 143, 335–336 (2010). 21029854 10.1016/j.cell.2010.10.021

[b28] Adam S., Polo S. E. & Almouzni G. Transcription recovery after DNA damage requires chromatin priming by the H3.3 histone chaperone HIRA. Cell 155, 94–106 (2013). 24074863 10.1016/j.cell.2013.08.029

[b29] Ray-Gallet D. . Dynamics of histone H3 deposition in vivo reveal a nucleosome gap-filling mechanism for H3.3 to maintain chromatin integrity. Mol. Cell 44, 928–941 (2011). 22195966 10.1016/j.molcel.2011.12.006

[b30] Schwab R. A., Blackford A. N. & Niedzwiedz W. ATR activation and replication fork restart are defective in FANCM-deficient cells. EMBO J. 29, 806–818 (2010). 20057355 10.1038/emboj.2009.385 PMC2829160

[b31] Conti C., Seiler J. A. & Pommier Y. The mammalian DNA replication elongation checkpoint: implication of Chk1 and relationship with origin firing as determined by single DNA molecule and single cell analyses. Cell Cycle 6, 2760–2767 (2007). 17986860 10.4161/cc.6.22.4932

[b32] Maya-Mendoza A., Petermann E., Gillespie D. A., Caldecott K. W. & Jackson D. A. Chk1 regulates the density of active replication origins during the vertebrate S phase. EMBO J. 26, 2719–2731 (2007). 17491592 10.1038/sj.emboj.7601714 PMC1888675

[b33] Episkopou H. . Alternative lengthening of telomeres is characterized by reduced compaction of telomeric chromatin. Nucleic Acids Res. 42, 4391–4405 (2014). 24500201 10.1093/nar/gku114 PMC3985679

[b34] Schwartzentruber J. . Driver mutations in histone H3.3 and chromatin remodelling genes in paediatric glioblastoma. Nature 482, 226–231 (2012). 22286061 10.1038/nature10833

[b35] Sfeir A. . Mammalian telomeres resemble fragile sites and require TRF1 for efficient replication. Cell 138, 90–103 (2009). 19596237 10.1016/j.cell.2009.06.021 PMC2723738

[b36] Duquette M. L., Handa P., Vincent J. A., Taylor A. F. & Maizels N. Intracellular transcription of G-rich DNAs induces formation of G-loops, novel structures containing G4 DNA. Genes Dev. 18, 1618–1629 (2004). 15231739 10.1101/gad.1200804 PMC443523

[b37] Arora R. . RNaseH1 regulates TERRA-telomeric DNA hybrids and telomere maintenance in ALT tumour cells. Nat. Commun. 5, 5220 (2014). 25330849 10.1038/ncomms6220 PMC4218956

[b38] Fan Q., Zhang F., Barrett B., Ren K. & Andreassen P. R. A role for monoubiquitinated FANCD2 at telomeres in ALT cells. Nucleic Acids Res. 37, 1740–1754 (2009). 19129235 10.1093/nar/gkn995 PMC2665210

[b39] Zeng S. . Telomere recombination requires the MUS81 endonuclease. Nat. Cell Biol. 11, 616–623 (2009). 19363487 10.1038/ncb1867 PMC2675667

[b40] Teng S. C., Chang J., McCowan B. & Zakian V. A. Telomerase-independent lengthening of yeast telomeres occurs by an abrupt Rad50p-dependent, Rif-inhibited recombinational process. Mol. Cell 6, 947–952 (2000). 11090632 10.1016/s1097-2765(05)00094-8

[b41] Ghosal G. & Muniyappa K. Saccharomyces cerevisiae Mre11 is a high-affinity G4 DNA-binding protein and a G-rich DNA-specific endonuclease: implications for replication of telomeric DNA. Nucleic Acids Res. 33, 4692–4703 (2005). 16116037 10.1093/nar/gki777 PMC1188515

[b42] Burrell R. A. . Replication stress links structural and numerical cancer chromosomal instability. Nature 494, 492–496 (2013). 23446422 10.1038/nature11935 PMC4636055

[b43] Harley C. B., Futcher A. B. & Greider C. W. Telomeres shorten during ageing of human fibroblasts. Nature 345, 458–460 (1990). 2342578 10.1038/345458a0

[b44] Ourliac-Garnier I. & Londono-Vallejo A. Telomere strand-specific length analysis by fluorescent in situ hybridization (Q-CO-FISH). Methods Mol. Biol. 735, 33–46 (2011). 21461809 10.1007/978-1-61779-092-8_4

[b45] Banaszynski L. A. . Hira-dependent histone H3.3 deposition facilitates PRC2 recruitment at developmental loci in ES cells. Cell 155, 107–120 (2013). 24074864 10.1016/j.cell.2013.08.061 PMC3838450

[b46] Bolte S. & Cordelieres F. P. A guided tour into subcellular colocalization analysis in light microscopy. J. Microsc. 224, 213–232 (2006). 17210054 10.1111/j.1365-2818.2006.01706.x

