# Supplementary material for: Suppression of the alternative lengthening of telomere pathway by the chromatin remodelling factor ATRX
Source: Nat Commun. 2015 Jul 6;6:7538. doi: 10.1038/ncomms8538 (PMC4501375; doi:10.1038/ncomms8538)
Supplement: Supplementary Information — Supplementary Figures 1-5 and Supplementary Table 1 [file ncomms8538-s1.pdf]

SUPPLEMENTARY INFORMATION

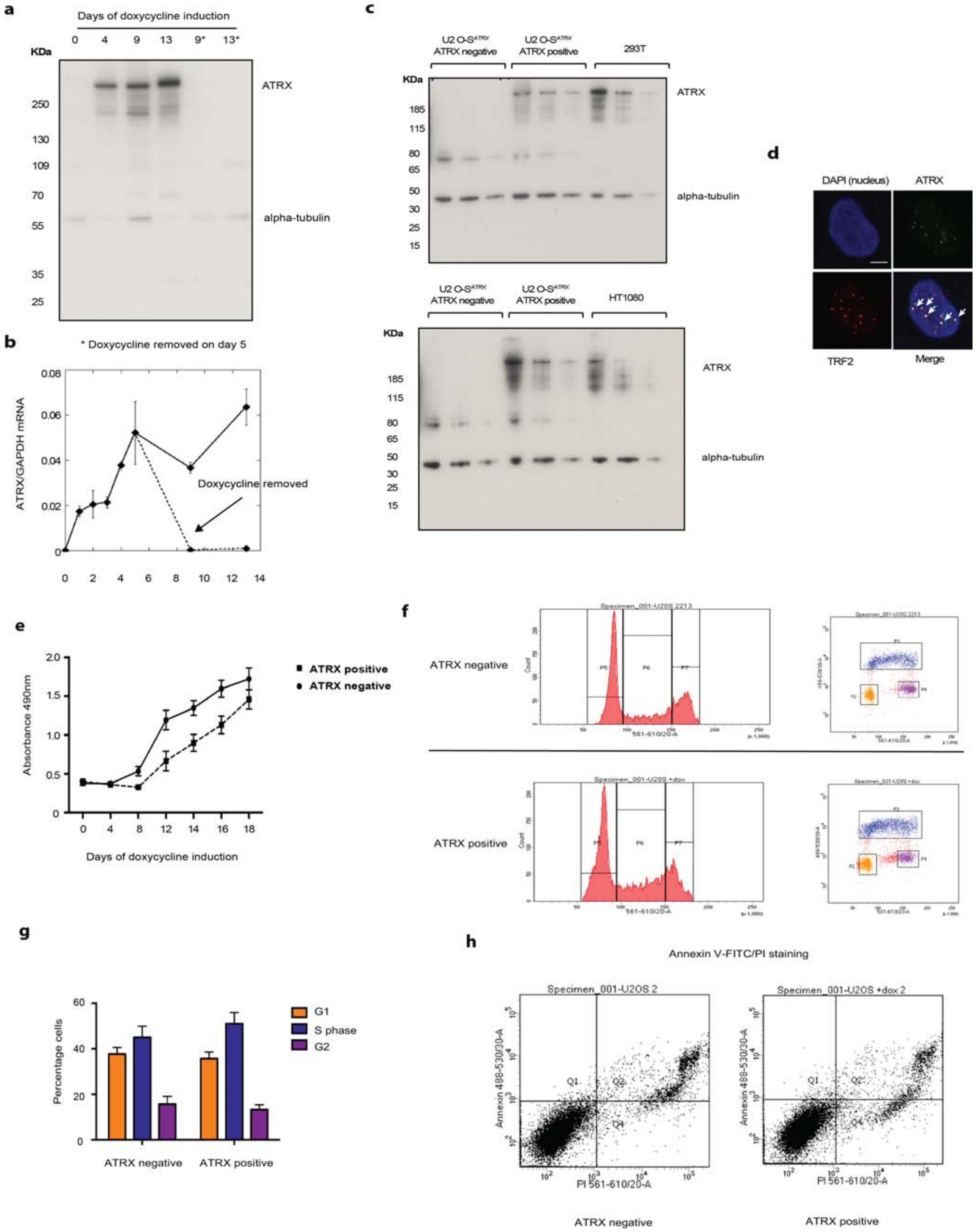

Supplementary Figure 1. Exogenously expressed ATRX represses the ALT pathway. (a) Immunoblot showing expression of ATRX in U-2 OS^{ATRX} cell line upon addition of 0.4 µg/ml doxycycline for the specified number of days. Alpha tubulin is shown as a loading control. (b) Reverse transcription PCR showing expression of ectopic ATRX mRNA in U-2 OS^{ATRX} cells upon addition of 0.4 µg/ml doxycycline for the specified number of days. All RNA levels are normalized to GAPDH transcript levels. (c) Immunoblot showing that ectopic ATRX expression levels in induced U-2 OS^{ATRX} fall within the range of endogenous ATRX expression of two control cell lines (293T and HT1080). Three decreasing dilutions of each cell extract have been loaded. Alpha tubulin is shown

as loading control. (d) Immunofluorescence showing ATRX and TRF2 co-localising in a U-2 OS^{ATRX} cell nucleus. A 10 µm scale marker is shown. (e) Cell cycle proliferation assay (MTS) in U-2 OS cells both in the presence and absence of 0.4 µg/ml doxycycline for the specified number of days. Absorbance at 490nm is proportional to the number of living cells in culture (f) Cell cycle of uninduced and induced U-2 OS cells (4 days 0.4 µg/ml doxycycline) was analysed by FACS. (g) In U-2 OS^{ATRX} cells expressing ATRX, a small but consistently reproducible increase of cells in S phase and a decrease of cells in G2/M. (h) Annexin V-FITC/PI staining FACs plots of U-2 OS cells with and without ectopic ATRX expression for 4 days. Annexin V staining is shown on the Y-axes with positive staining indicating apoptopic cells. Cells were counterstained with propidium iodide (X-axes) to discriminate necrotic/dead cells from apoptotic cells.

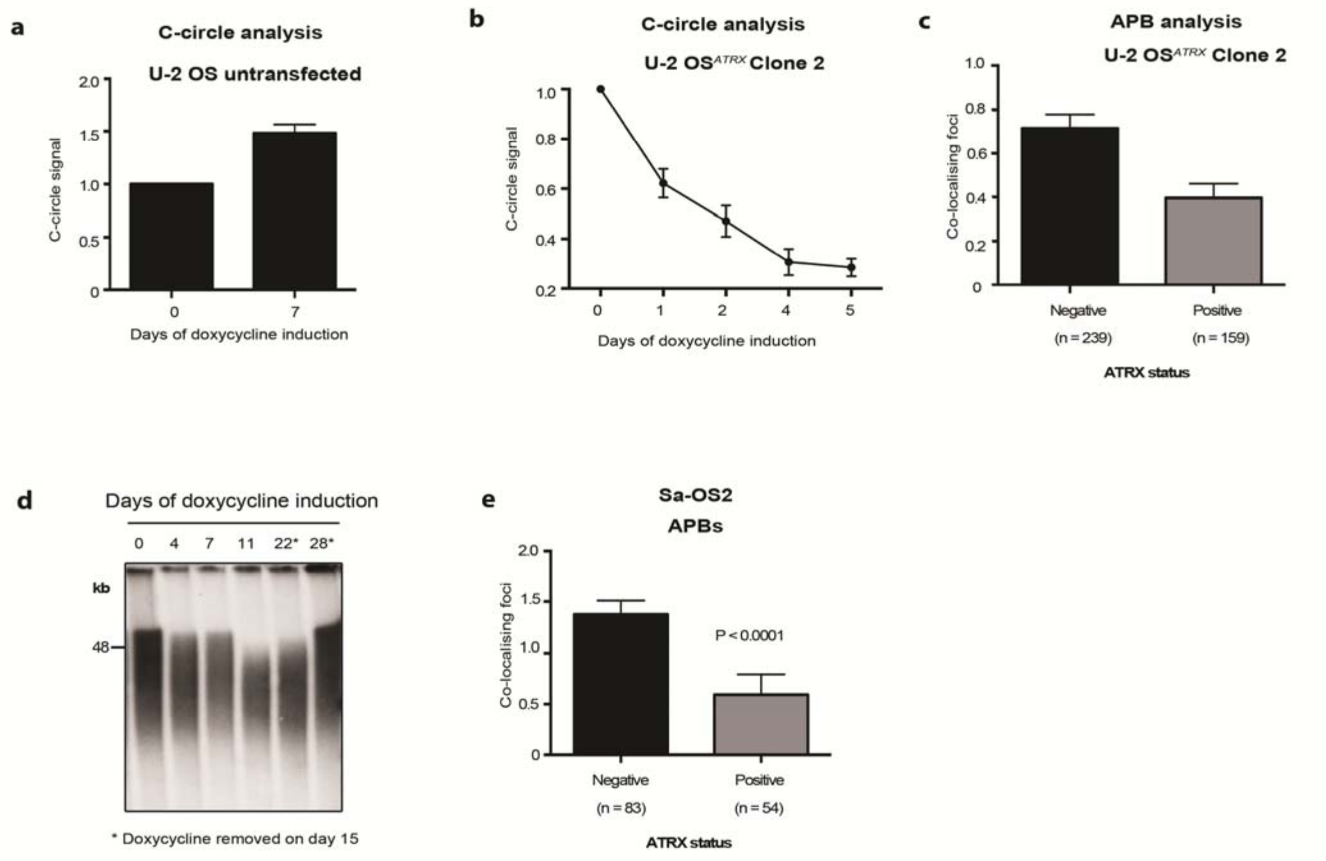

Supplementary Figure 2. (a) C-circle assay of untransfected U-2 OS cells upon addition of 0.4 µg/ml doxycycline for 7 days. (b) C-circle assay showing loss of C-circles upon ectopic expression of ATRX in a second independently derived U-2 OS^{ATRX} clone. (c) APB analysis in the second independently derived U-2 OS^{ATRX} clone shows a decrease in co-localising foci. (d) Terminal restriction fragment length analysis using a ^{32}P-labelled TTAGGG probe showing progressive telomere shortening upon expression of ATRX and recovery upon removal of doxycycline. Re-lengthening of telomeres was observed upon removal of doxycycline at day 15 (*) (e) Transient transfection of ATRX into Sa-OS2 cells leads to a reduction of C-circles.

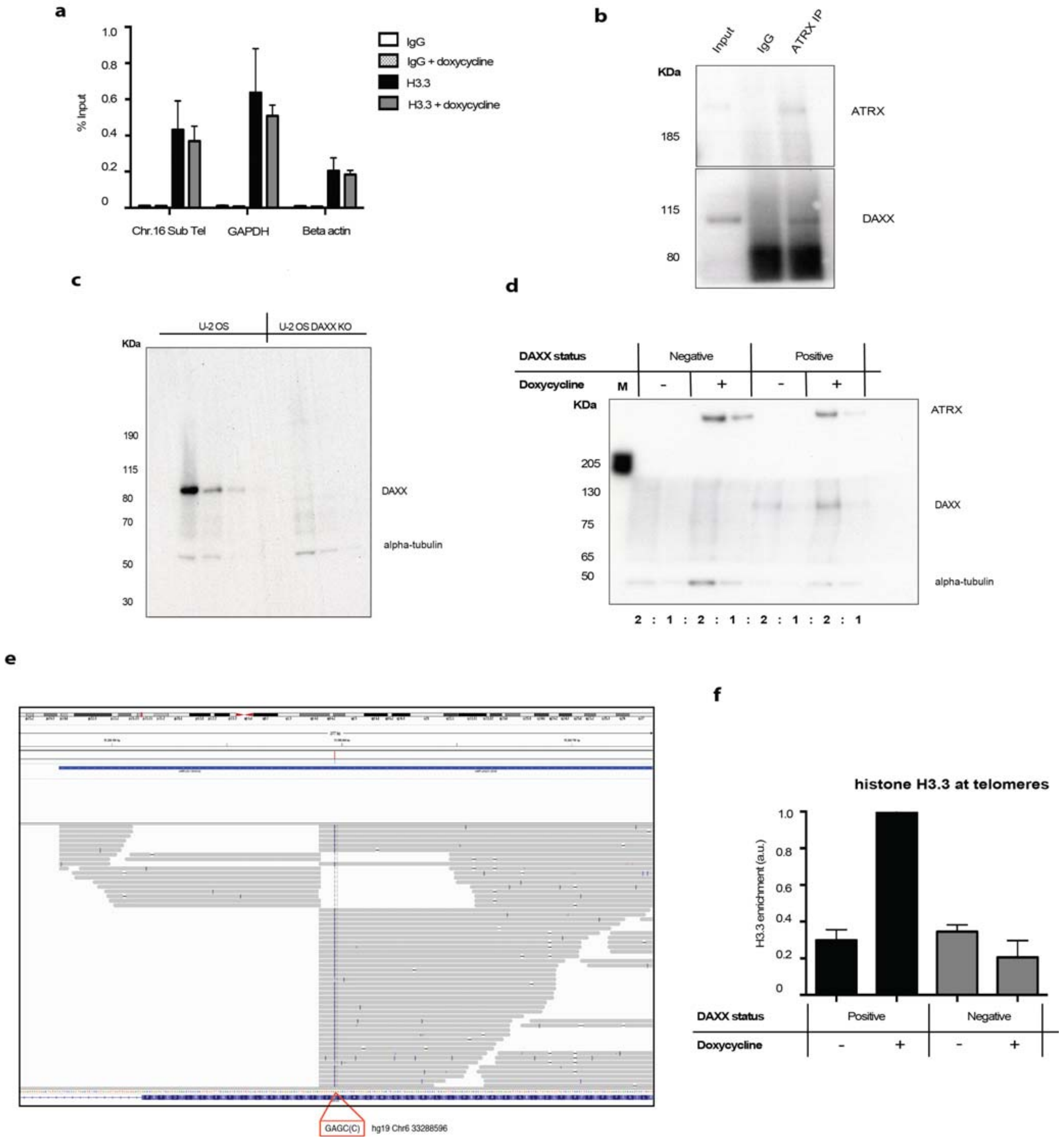

Supplementary Figure 3. (a) ChIP assay showing levels of histone H3.3 are unaffected at the subtelomere of chromosome 16, GAPDH and beta actin upon ATRX expression. Graph shows average of three biological replicates. Error bars denote S.E.M. (b) DAXX immunoprecipitation from U-2 OS^{ATRX} nuclear extract in the presence of ectopic ATRX, using an ATRX-specific antibody (c) Immunoblot showing serial dilutions of a U-2 OS^{ATRX} clone in which DAXX was successfully knocked-out. Alpha tubulin is shown as a loading control. (d) Western blot confirming inducible ATRX expression in the DAXX knockout clone obtained by the Zinc Finger Endonucleases (ZFNs) genome editing approach. Cells were treated for 4 days with 0.4 μg/ml doxycycline M=protein size marker. (e) The presence of two heterozygous genomic insertions (GAGC or GAGCC) immediately adjacent to the ZFN cut site was revealed by Ion Torrent sequencing. (f) Chromatin immunoprecipitation in uninduced and induced (4 days 0.4 μg/ml doxycycline) U-2 OS^{ATRX} cells shows that histone H3.3 enrichment at telomeres upon ATRX expression is lost in the absence of DAXX. Enrichment is shown relative to ATRX/DAXX positive telomeric histone H3.3 levels.

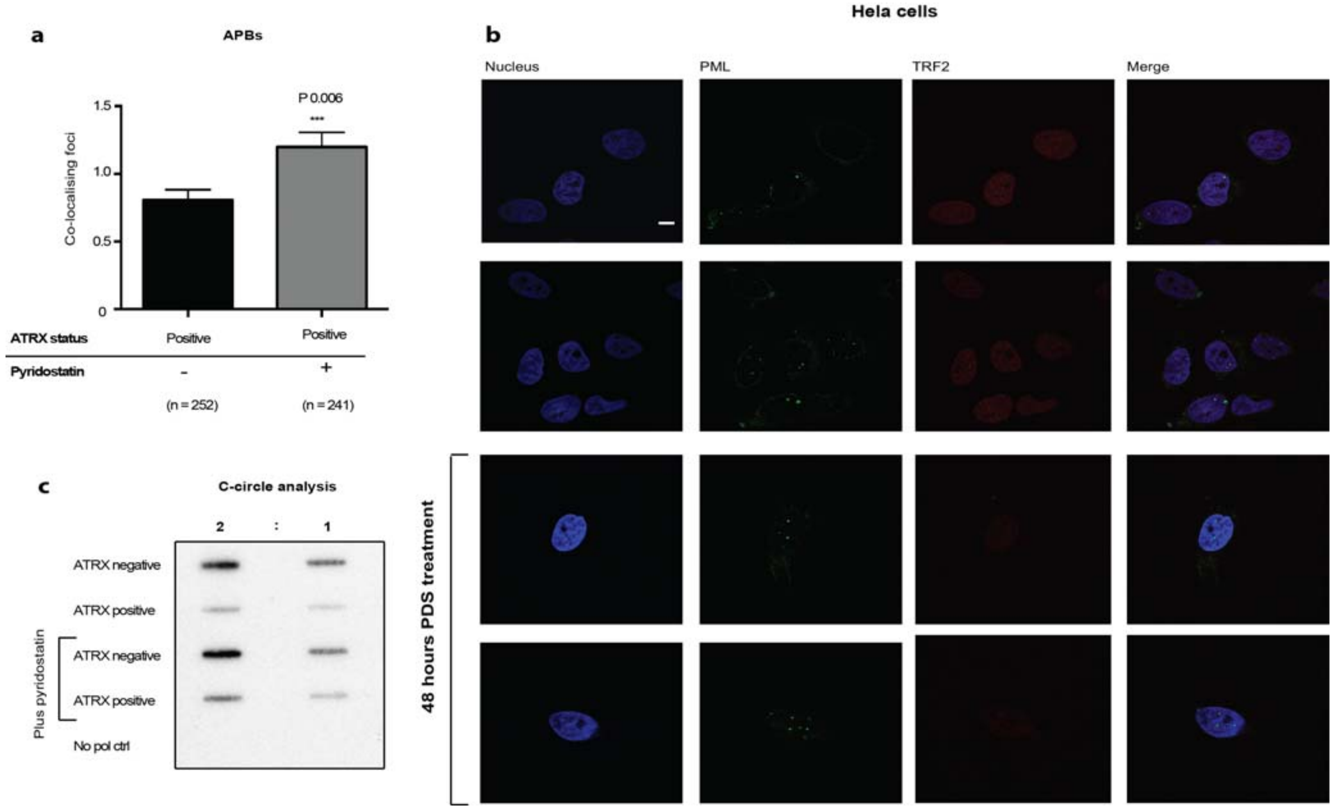

Supplementary Figure 4. Treatment with a G-quadruplex stabilizing ligand impedes ability of ATRX to suppress ALT. (a) Quantitation of APB analysis in induced (4 days 0.4 µg/ml doxycycline) U-2 OS^{ATRX} cells in the presence and absence of 20 µM PDS for 48 hours. Number of cells scored is shown in parentheses and statistical significance was determined using a Mann Whitney test. (b) Representative immunofluorescence images of HeLa cells showing the absence of detectable APB bodies following treatment with 20 µM PDS for 48 hours. (c) Representative slot blot of C-circle assay in the presence and absence of PDS and ATRX (with or without 4 days treatment with 0.4 µg/ml doxycycline).

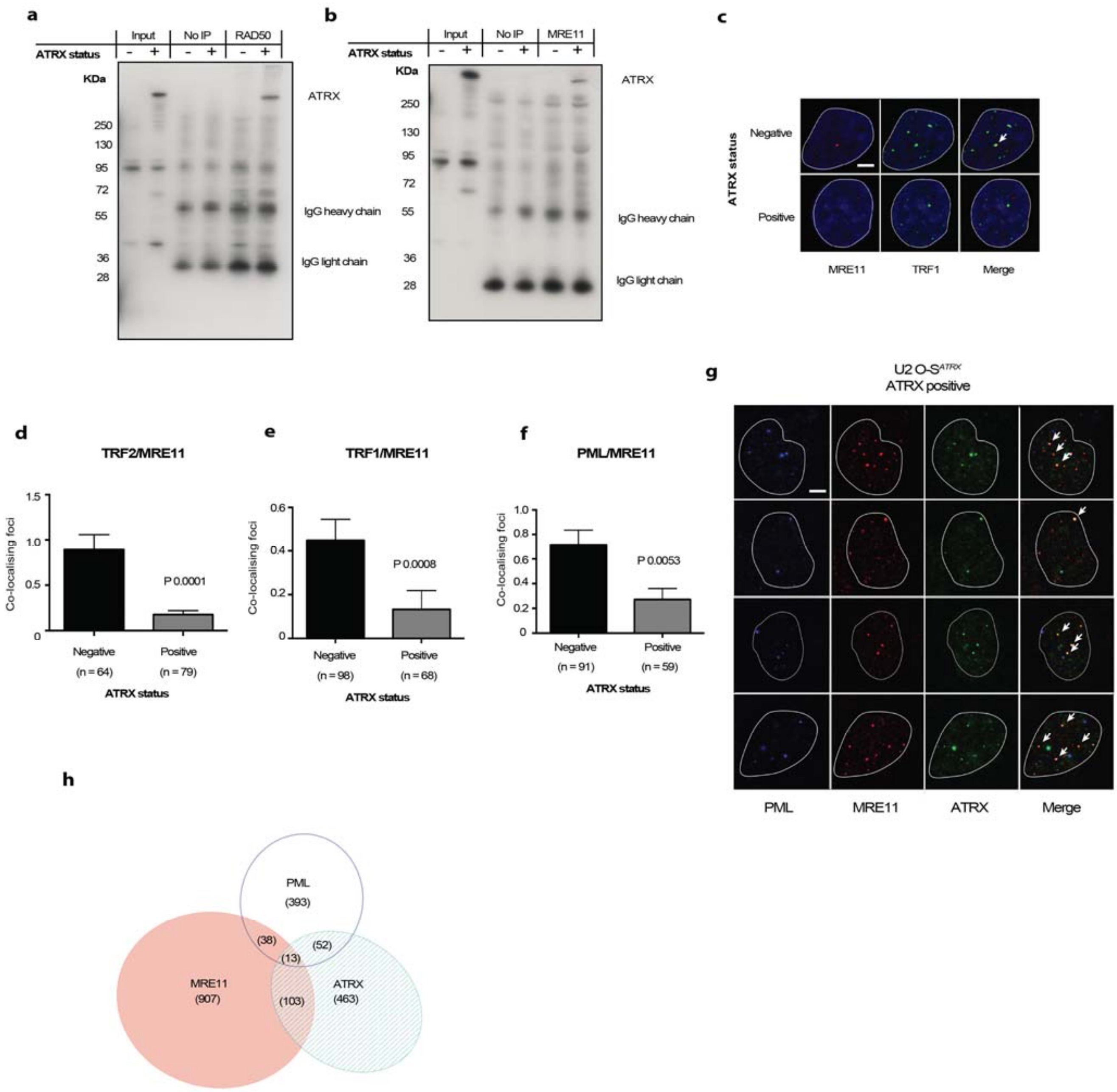

Supplementary Figure 5. Exogenously expressed ATRX in U-2 OS^{ATRX} cells interacts with the MRN complex and alters its localization. (a) and (b) Nuclear extracts from U-2 OS^{ATRX} cell line with and without exogenously expressed ATRX were immunoprecipitated (IP'd) with both (a) RAD50 and (b) MRE11 antibodies. For no IP beads, blocked beads were added without prior antibody coupling. The presence of ATRX with immunoisolated interaction partners was assessed by Western blotting. (c) Immunofluorescence showing loss in co-localization between MRE11 and telomeric marker TRF1 upon ATRX expression (1 month treatment with 0.4 µg/ml doxycycline); a 10 µm scale marker is shown. (d) Quantitation of APB analysis showing loss in co-localization between MRE11 and TRF1, (e) TRF2 and (f) PML following 1 month 0.4 µg/ml doxycycline treatment. (g) Representative image showing 3-way co-localisation analysis between PML, MRE11 and ATRX. A 10 µm scale marker is shown. (h) Venn diagram showing co-localisation between MRE11 and PML decreases upon ATRX expression, and ATRX co-localisation with MRE11 is generally distinct from PML bodies. Total number of foci is shown in parentheses. Venn diagrams were plotted using eulerdiagrams.

Supplementary Table 1: Primer sequences

**hGAPDF/R**	ACCTGTGCTCCCACTCCTGATTTC	TGCCAAGTTGCCTGTCCTTCC
**16p Sub-telomere**	CCTCGCCTTGCCTTGGGAG	CGGTTCAGTGTGGAAAATGGGAAAC
**Beta actin**	TCCCTGGAGAAGAGCTACGA	AGGAGGGAAGGGTGGAAGAG
